# Clustering care pathways of people with alcohol dependence using a data linkage of routine data in Bremen, Germany

**DOI:** 10.1186/s12916-024-03438-4

**Published:** 2024-05-30

**Authors:** Justin Möckl, Jakob Manthey, Monika Murawski, Christina Lindemann, Bernd Schulte, Jens Reimer, Oliver Pogarell, Ludwig Kraus

**Affiliations:** 1 Department of Epidemiology and Diagnostics, IFT Institut Für Therapieforschung, Centre for Mental Health and Addiction Research, Munich, Germany; 2 Department of Psychiatry and Psychotherapy, University Hospital, LMU Ludwig-Maximilians-Universität Munich, Munich, Germany; 3 Center for Interdisciplinary Addiction Research, Department of Psychiatry and Psychotherapy, University Medical Center Hamburg-Eppendorf, Hamburg, Germany; 4 Department of Psychiatry, Medical Faculty, University of Leipzig, Leipzig, Germany; 5 Department of Medical Psychology, Center for Health Care Research, University Medical Center Hamburg-Eppendorf, Hamburg, Deutschland; 6 Zentrum Für Psychosoziale Medizin, Klinikum Itzehoe, Itzehoe, Germany; 7 Department of Public Health Sciences, Centre for Social Research On Alcohol and Drugs, Stockholm University, Stockholm, Sweden; 8 Institute of Psychology, ELTE Eötvös Loránd University, Budapest, Hungary

**Keywords:** Alcohol dependence, Treatment utilization, Data linkage, Routine data, State sequence analysis

## Abstract

**Background:**

Although many individuals with alcohol dependence (AD) are recognized in the German healthcare system, only a few utilize addiction-specific treatment services. Those who enter treatment are not well characterized regarding their prospective pathways through the highly fragmented German healthcare system. This paper aims to (1) identify typical care pathways of patients with AD and their adherence to treatment guidelines and (2) explore the characteristics of these patients using routine data from different healthcare sectors.

**Methods:**

We linked routinely collected register data of individuals with a documented alcohol-related diagnosis in the federal state of Bremen, Germany, in 2016/2017 and their addiction-specific health care: two statutory health insurance funds (outpatient pharmacotherapy for relapse prevention and inpatient episodes due to AD with and without qualified withdrawal treatment (QWT)), the German Pension Insurance (rehabilitation treatment) and a group of communal hospitals (outpatient addiction care). Individual care pathways of five different daily states of utilized addiction-specific treatment following an index inpatient admission due to AD were analyzed using state sequence analysis and cluster analysis. The follow-up time was 307 days (10 months). Individuals of the clustered pathways were compared concerning current treatment recommendations (1: QWT followed by postacute treatment; 2: time between QWT and rehabilitation). Patients’ characteristics not considered during the cluster analysis (sex, age, nationality, comorbidity, and outpatient addiction care) were then compared using a multinomial logistic regression.

**Results:**

The analysis of 518 individual sequences resulted in the identification of four pathway clusters differing in their utilization of acute and postacute treatment. Most did not utilize subsequent addiction-specific treatment after their index inpatient episode (*n* = 276) or had several inpatient episodes or QWT without postacute treatment (*n* = 205). Two small clusters contained pathways either starting rehabilitation (*n* = 26) or pharmacotherapy after the index episode (*n* = 11). Overall, only 9.3% utilized postacute treatment as recommended.

**Conclusions:**

A concern besides the generally low utilization of addiction-specific treatment is the implementation of postacute treatments for individuals after QWT.

**Supplementary Information:**

The online version contains supplementary material available at 10.1186/s12916-024-03438-4.

## Background

The consumption of alcohol was responsible for an estimated 5.3% of all global deaths and 5.1% of all disability-adjusted life years in 2016 [1]. Germany is considered a high-consumption country, even though per capita consumption of pure alcohol per person aged older than 15 years has decreased over the last 20 years from 12.0 l in 2000 to 10.0 l in 2021 [2]. Mental and behavioral disorders due to alcohol (F10.X) according to the International Classification of Diseases (ICD-10) were the fourth most frequent main diagnoses for inpatient hospitalization in 2021 [3]. Although an estimated 35% of people with alcohol dependence were diagnosed in medical health care settings in Germany in 2012, only 16% received addiction-specific care services, indicating a significant treatment gap [4].

In general, the treatment of alcohol dependence in Germany occurs in a highly fragmented healthcare system, which is based on mandatory health insurance using either public statutory health insurance or private health insurance. Different actors are responsible for the reimbursement of costs for addiction-specific treatment and care services. While outpatient addiction care services are primarily financed by communes, acute treatments, such as withdrawal management, are covered by health insurance funds. Health insurance funds can also cover the cost of some postacute treatments, such as outpatient pharmacotherapy. A rehabilitation treatment, either inpatient or outpatient, is nevertheless financed in most cases by the German Pension Insurance (Deutsche Rentenversicherung (DRV)), but only if certain requirements are met [5]. These requirements, among others, are not being a pensioner or a civil servant and having paid for the insurance for at least six months over the past two years.

A comprehensive discussion and practical recommendations based on available evidence of multiple treatment options for risky, harmful, and dependent alcohol use were elaborated in the current German S3 guidelines on “Screening, diagnosis, and treatment of alcohol-related disorders” [5]. The recommended treatment for people with alcohol dependence consists of acute treatment (e.g., withdrawal treatment including detoxification) followed — ideally seamlessly — by postacute treatment, either by using pharmacotherapy for relapse prevention, rehabilitation treatment, or other postacute treatments, such as psychotherapy, and inpatient/outpatient psychiatric care [5]. Depending on the severity of withdrawal, the number of prior withdrawal treatments, and the social integration of the patient, withdrawal treatment can take place either in inpatient or outpatient settings. Guidelines recommend providing a so-called Qualified Withdrawal Treatment (QWT), which describes a German-specific term for an extended withdrawal treatment program (generally three weeks) including psychosocial interventions [5]. Detoxification, i.e., controlling and reducing alcohol withdrawal symptoms as well as any neurological or physical symptoms (e.g., epileptic seizures or delirium tremens), is but one component during QWT. Additionally, treatment of the underlying dependence is initiated while considering psychological and somatic concomitant and secondary diseases. The goals are to increase motivation to seek further help and more specific treatments (e.g., addiction rehabilitation) and to establish contact with the regional support system (e.g., psychotherapy, self-help) [6]. Despite clear recommendations, a recent study indicated that QWT for people with severe alcohol use disorders is considerably underutilized [7].

The aim of this study was the exploratory analysis of empirical addiction-specific care pathways of patients with alcohol dependence using linked data of different payers in addiction-specific care services and treatments. To this end, a state sequence analysis was performed. State Sequence Analysis was originally developed to analyze educational lifetime trajectories in social sciences but has recently been adapted to epidemiological analyses of care pathways for different conditions [8–10]. Here, individual treatment states constitute sequences that represent individual care pathways. By clustering these sequences, typical pathways as well as their adherence to current guideline recommendations were elaborated. Finally, the resulting clusters were compared concerning sociodemographic characteristics and their addiction-specific care utilization.

## Methods

### Data

Three routine data sources collected within the project “Implementation and Evaluation of the S3 Guideline on Screening, Diagnosis, and Treatment of Alcohol-Related Disorders” (IMPELA) were used. The data sources contained individual information for persons aged 16 years or older living in the northern German federal city-state of Bremen between 2016 and 2017. The overall sample comprised all people with a main or secondary diagnosis of mental and behavioral disorders due to alcohol (F10.X) or another fully alcohol-attributable diagnosis (for a detailed list see Additional file 1: Table S1). The diagnosis was documented in at least one of three used data sources: (a) two statutory health insurance funds (AOK Bremen/Bremerhaven and hkk), (b) the regional German Pension Insurance (DRV Oldenburg/Bremen), and (c) the outpatient addiction care of the municipal clinic association Gesundheit-Nord [11]. The three data sources included information on different addiction-specific treatments and care services, i.e., billed treatments in both inpatient and outpatient settings (a: statutory health insurance funds), rehabilitation services in inpatient, outpatient, or both settings (b: pension insurance), and visits to outpatient addiction care (c: municipal clinic association). Data from these sources were linked at the individual level [12]. In the state sequence analysis, only sequences of people with information from the statutory health insurance funds were considered.

The population insured with the two statutory health insurance funds represents approximately 50% of the total population in Bremen (307,245 out of 584,516) and shows a similar distribution of age and gender compared to the total population in Bremen (For a more detailed description of the total sample see 13). The overall sample with information from the statutory health insurance funds comprised 10,507 individuals. Individuals who were insured in both funds at the same time (*n* = 9), who were not insured in one of these two funds for more than 60 days (*n* = 338) or with recorded death (*n* = 82) were excluded. An index event was defined to ensure a homogenous and comparable sample. Therefore, only pathways starting with an inpatient episode with a main diagnosis of alcohol dependence (F10.2) or withdrawal syndrome (F10.3–4) (*n* = 973) were included if they had no preceding inpatient episode of the same kind for at least 60 days prior and had data available for at least 10 months of follow-up time (*n* = 518). The index event could contain QWT. The 60 days served as a kind of wash-out period to ensure a similar baseline for the individual pathways while allowing for a follow-up period of at least 10 months (307 days). As the data were restricted to two calendar years and most first episodes already took place in the first four months of 2016, there was little room for alternative specifications of wash-out and follow-up periods. The distribution of the months in which the first inpatient episode took place is shown for the total sample in the appendix (see Additional file 1: Fig. S1). As the allowed 60 missing insurance days within 2016/2017 could fall into the observation period, a sensitivity analysis was done excluding cases with missing insurance days in the observation period (*n* = 16).

### Addiction-specific care services and treatments

Several addiction-specific treatments and care services in different settings were identified. For each inpatient hospital episode paid by the statutory insurance fund (a), ICD-10 diagnoses (main, secondary), date of admission and discharge, and administered procedures by assigned Surgery- and Procedure-Codes (Operationen- und Prozeduren-Schlüssel (OPS codes)) were documented. QWT could be identified in both somatic (OPS code 8–985) and psychiatric wards (OPS code 9–647). Since OPS codes are not substance specific, a main diagnosis for alcohol dependence or withdrawal state (F10.2–4) or any F10.X diagnosis with alcohol dependence (F10.2) as a secondary diagnosis for the specific inpatient episode had to be present. Detoxification not within a QWT is not specifically coded. The duration of each inpatient episode was calculated by the date of admission and discharge.

For treatments in the outpatient setting paid by the statutory insurance fund (a), diagnoses are billed and documented quarterly. Medication is documented by the pharmaceutical registration numbers (Pharmazentralnummer (PZN)), as well as the date of the prescription. Using this number and the corresponding anatomical-therapeutic-chemical classification (ATC), we identified medications specifically prescribed for drug relapse prevention (ATC code: N07BB). The assignment of registration numbers with ATC codes was based on the drug master file of the German Drug Index of the Scientific Institute of the AOK (Wissenschaftliches Institut der AOK (WIdO)) as of September 2017. For every drug, an exposure window of 90 days after the subscription was defined. Using the data from the regional German Pension Insurance (b), specific dates of rehabilitation treatments in different treatment settings (inpatient, outpatient, both) could be identified. Additionally, the number of visits to outpatient addiction care services of the communal hospital group Gesundheit-Nord (c) could be identified by visit dates with a documented F10.2 diagnosis.

Concerning treatment guideline adherence, the utilization of QWTs as well as the timing of postacute treatment after QWT were analyzed. Therefore, the duration in days between the end of a QWT and the onset of a rehabilitation treatment was calculated based on the admission date of the rehabilitation and the discharge date of the nearest QWT.

### Pathway construction

First, an alphabet of all possible states must be defined. States were defined as having used one of the explained addiction-specific treatments. Attending outpatient addiction care of the communal hospital group was not counted as a treatment. This resulted in the following alphabet containing five states of addiction-specific treatments:

A. No addiction-specific treatment
B. Outpatient pharmacotherapy for relapse prevention
C. Rehabilitation
D. Inpatient episode due to alcohol dependence (F10.2–4) incl. QWT
E. Inpatient episode due to alcohol dependence (F10.2–4)

For each individual, a sequence of 307 days, with each day containing one of the states defined in the alphabet, was created. The number of days in the follow-up period always included the index event to ensure the cluster analysis could take the different lengths of the index event into account. These sequences represent the individual addiction-specific care pathways. If another of the defined treatments, i.e., C, D, or E, which contain clear admission and discharge dates, was present within the exposure window of B, treatment C, D, or E was coded instead of B for this specific time frame. If the discharge date was still within the 90-day exposure window of B, B was again coded until the end of the 90-day exposure window. Additionally, it was assumed that if B had already started before the index event, it was resumed afterwards.

### Cluster analysis of pathways

After defining the state sequences, these individual pathways were clustered. While patients who did not utilize further addiction-specific treatments despite their index episode were set as the reference cluster (Cluster 0), the remaining pathways with at least one further addiction-specific treatment, i.e., states B–E, were clustered to typical care pathways. A dissimilarity matrix was created using a dissimilarity measure called the longest common subsequence (lcs) [14, 15]. This measure defines the similarity between sequences (*x* and *y*) by using the length of common elements (states) occurring in the same order (lcs(*x*,*y*)). The distance *d* between sequences *x* and *y* is then defined as

$$d=l\left(x\right)+l\left(y\right)-2(\text{lcs}\left(x,y\right))$$

where *l* denotes the length of the sequence. The distance, therefore, is based on the elements not part of the longest common subsequence.

Based on this dissimilarity measure, clustering techniques were used to group sequences and identify typical addiction-specific care pathways. Hierarchical clustering, as well as partitioning around medoids (PAM), also called k-medoids clustering, were calculated, and then compared. These two methods differ in how clustering is performed. Hierarchical clustering is a bottom-up approach that starts with clustering every sequence as one single cluster and then locally minimizes differences by merging clusters until only one cluster is left. The ward criterion was used, which minimizes the residual variance [16]. K-medoids clustering offers an advantage over hierarchical clustering through the optimization of a global parameter instead of a local parameter, as its aim is to identify the best representatives (medoids) for a given number of groups [16]. These medoids are defined as having the smallest weighted sum of distances from other observations in the group. A disadvantage of this method is that the starting points for the optimization and the number of clusters must be defined in advance. To find the optimal solution, the hierarchical cluster solution was used as a starting point for k-medoids clustering using different numbers of clusters (here five).

For both clustering techniques, the best number of clusters must be identified. This was done by calculating the average silhouette width (range: − 1–1) and Hubert’s C (range: 0–1). The average silhouette width allows for the comparison of different clustering solutions in terms of coherence of assignment by between-group differences and within-group homogeneity (the higher, the better) and Hubert’s C, which indicates the gap between the present solution and the best solution theoretically possible (the lower, the better) [16]. Finally, the solution with the highest average silhouette width, lowest Hubert’s C, and highest interpretability will be presented in the following.

Typical treatment pathways were constructed by selecting the 10 most representative sequences using their neighborhood density [17]. Using the distance matrix, a representative score was calculated based on the number of sequences in the neighborhood of each sequence, meaning that their distance was within a selected threshold. This threshold (neighborhood radius) was set at 10% of the maximum theoretical distance between two sequences. The coverage score corresponds to the number of sequences in the neighborhood of a representative sequence, and the total coverage corresponds to the number of sequences with a representative in their neighborhood [18]. The data were analyzed using the “TraMineR,” “WeightedCluster,” “cluster,” and “comorbidity” packages in R version 4.2.2 [19]. The R script of the analyses is part of the appendix (see Additional file 2).

### Patient characteristics

Comorbidities can affect health care utilization and might also influence which addiction-specific care service should be or can be used. To control for different levels of comorbidity at the start of the pathway, the Walraven-Elixhauser comorbidity score was calculated using all inpatient diagnoses, main and secondary, in the index episode and 60 days prior. Another study [20] has previously used this score in a similar manner, and it theoretically ranges from − 19 to 89 [21]. The score incorporates the association of different comorbidity groups with death in the hospital, in which higher scores signify a more severe level of comorbidity [21].

General and addiction-specific hospitalizations in the follow-up period were compared by the days spent in inpatient episodes and the share of hospitalizations due to alcohol dependence or withdrawal (F10.2–4) as the main diagnosis. Additionally, visits to outpatient addiction care and the time spent in inpatient episodes, including QWTs, were compared across clusters. Comparisons for categorical variables were performed using *χ* ^2^ tests and Fisher’s exact tests when cell sizes were smaller than five. Metric variables were compared using ANOVA when assuming normality and the Kruskal‒Wallis rank sum test when assuming nonnormality. Additionally, predictors of cluster membership (dependent variable) were calculated by a multinomial regression with the following independent variables: sex (male/female), age (centered), nationality (German/Non-German), Walraven-Elixhauser comorbidity score, inpatient episode before index event (no main diagnosis of alcohol dependence or withdrawal), the use of outpatient addiction care of the communal hospital group both in the 60 days before the index event (yes/no) and in the 10 months following the index event (yes/no).

## Results

### Cluster analysis

The analyzed sample comprised 518 patients with individual sequences of addiction-specific care after their index inpatient episode. In the total sample, the most often used addiction-specific care service was QWT (29.9%) and visiting outpatient addiction care within the follow-up period (11.6%). Furthermore, 9.3% utilized a postacute treatment, i.e., pharmacotherapy for relapse prevention (3.5%) or rehabilitation (6.4%). Just over half were assigned to the predefined Cluster 0 (*n* = 276). Cluster analysis (k-medoids clustering; average silhouette width 0.59, and Hubert’s C 0.05) resulted in Cluster 1 (*n* = 205), Cluster 2 (*n* = 26), and Cluster 3 (*n* = 11). The sociodemographic characteristics and utilized care services of the sample in total and by cluster are presented in Table 1. For a graphical representation of all individual sequences, see Additional file 1: Fig. S2.

**Table 1:** Summary statistics of patients with an index episode by clusters

		**Total**	**Addiction-specific care after the index episode**						
			**Cluster 0**		**Cluster 1**	**Cluster 2**		**Cluster 3**	***P*** ** value**
.	*n*	518	276		205	26		11	
Female	%	23.7	26.4		22.9	3.8		18.2	.044
German nationality	%	90.3	91.2		89.2	88.5		90.9	.806
Age	Mean (SD)	50.1 (11.7)	50.4 (12.2)		50.2 (11.3)	47.0 (9.9)		49.5 (8.9)	.546
Comorbidity score	Median [IQR]	0.0 [− 2.8, 5.0]	0.0 [− 3.0, 5.0]		0.0 [− 1.0, 5.0]	0.0 [0.0, 10.3]		0.0 [− 2.5, 5.0]	.593
Hospital days within the follow-up period	Median [IQR]	21.0 [10.0, 39.0]	12.0 [7.0, 21.0]		35.0 [21.0, 56.0]	38.5 [28.0, 62.0]		29.0 [16.5, 42.0]	< .001
% hospital days due to alcohol dependence^1^	Median [IQR]	100.0 [72.6, 100.0]	100.0 [67.6, 100.0]		97.0 [73.7, 100.0]	95.3 [88.0, 100.0]		100.0 [93.7, 100.0]	.088
**Alcohol-specific care/treatment services**									
OAC^2^ prior to index episode	%	5.8	2.9		9.8	7.7	0.0		.012
OAC^2^ follow-up period	%	11.6	4.7		18.5	30.8	9.1		< .001
OAC^2^ follow-up period: total visits	Median [IQR]	2.0 [1.0, 5.0]	1.0 [1.0, 2.0]		2.5 [1.0, 4.8]	4.5 [2.8, 5.8]	2.0 [2.0, 2.0]		.091
Pharmacotherapy	%	3.5	0.0		1.5	15.4	100.0		< .001
Qualified withdrawal treatment (QWT)	%	29.9	18.8	41.0		61.5	27.3		< .001
Number of started QWT	Median [IQR]	1.0 [1.0, 1.0]	1.0 [1.0, 1.0]	1.0 [1.0, 2.0]		1.0 [1.0, 1.0]	1.0 [1.0, 1.0]		< .001
Postacute treatment	%	9.3	0.0	5.9		96.2	100.0		< .001
QWT before postacute treatment	%	3.9	0.0	1.0		57.7	27.3		< .001
Started rehabilitation	%	6.4	0.0	4.4		92.3	0.0		< .001
QWT before rehab	%	3.1	0.0	1.0		53.8	0.0		< .001
Days between QWT and rehab	Median [IQR]	22.5 [13.5, 38.5]	-	59.5 [40.3, 78.8]		22.5 [10.5, 29.8]	-		.427

*SD *Standard deviation, *IQR *Interquartile range; when nonnormality was assumed, the median instead of the mean is presented, and the *p* value of the Kruskal‒Wallis rank sum test instead of a *t* test. Variables, where percentages are presented, were compared with a *χ* ^2^-test and Fisher’s exact test for the comparison of the clusters when cell sizes were small. ^1^Main diagnosis of dependence F10.2 or withdrawal F10.3–4.^2^ *OAC*, outpatient addiction care

In Cluster 0 (“No further treatment”), every fifth person (18.8%, see Table 1) utilized a QWT in their index episode, and 4.7% visited outpatient addiction care within the follow-up period. Other than the index episode and outpatient addiction care, no further addiction-specific treatment was used. In the median, the number of days in hospital in the follow-up period was 12 days.

Cluster 1 (“No (seamless) postacute treatment”) shows a more frequent usage of QWT (41.0%) but a low utilization of rehabilitation treatment (4.4%) or pharmacotherapy (1.5%) as postacute treatment. It shows the second highest number of days spent in hospital (median: 35.0), the highest number of QWTs (third quartile: 2), and the second lowest share of postacute treatments (5.9%). If QWT was utilized before rehabilitation treatment (1.0%), the median waiting time was 59.5 days.

Pathways in Cluster 2 (“Rehabilitation”) mainly represented rehabilitation as postacute treatment (92.3%). Some patients also used pharmacotherapy (15.4%) for relapse prevention. This cluster shows the highest number of days spent in the hospital (median: 38.5). If a rehabilitation treatment followed a QWT (57.7%), the median days between the end of QWT and the onset of rehabilitation were less than 23 days (for a plot of the distribution by clusters, see Additional file 1: Fig. S3).

The remaining Cluster 3 (“Pharmacotherapy”) contained the fewest patients (*n* = 11). All of them started pharmacotherapy treatment. This cluster has the second lowest median number of hospital days (29 days), and the second lowest share of people using QWT (27.3%).

### Typical pathways

**Fig. 1:**
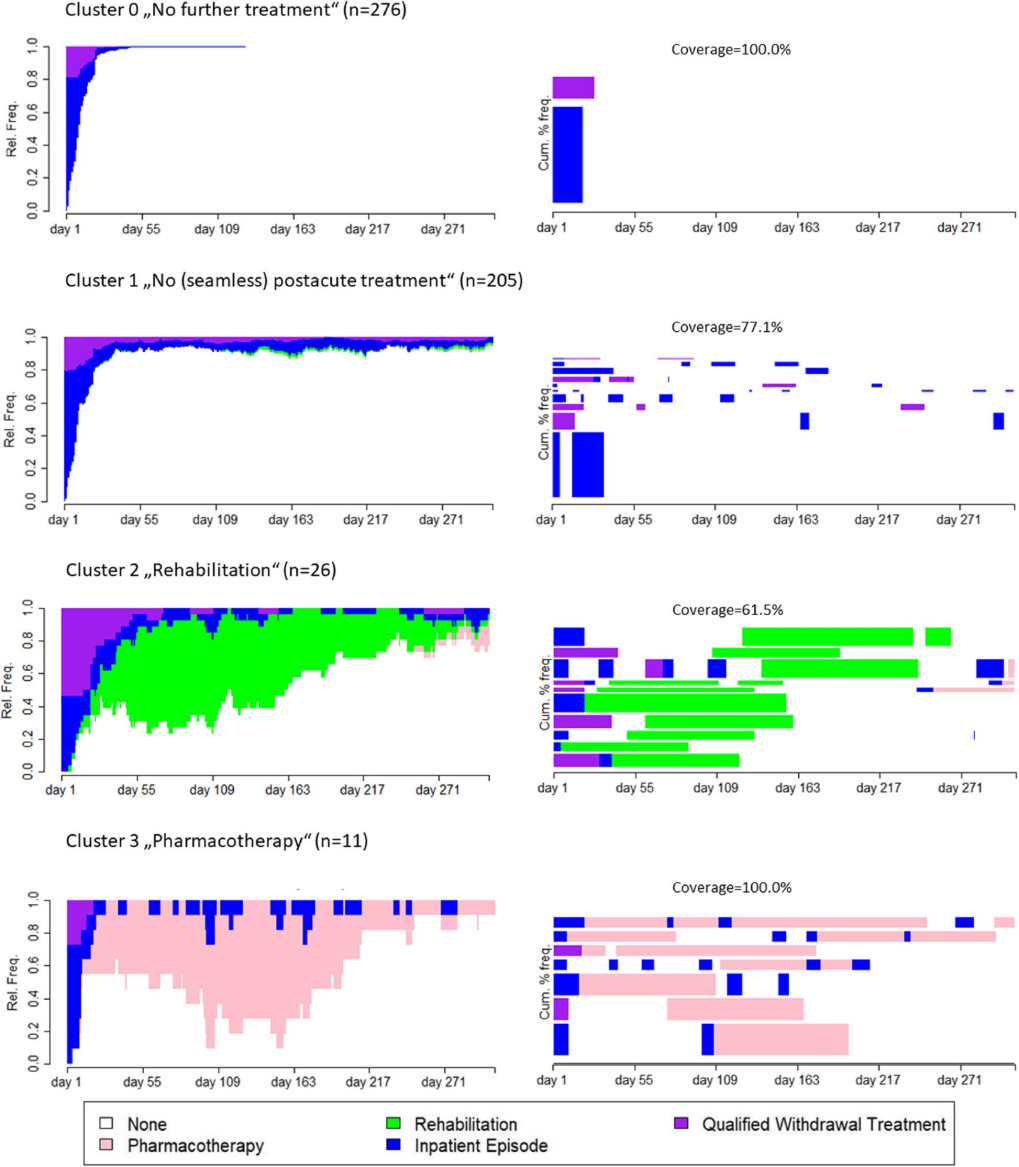
Relative frequency of addiction-specific care services after the index episode and typical pathways by cluster Notes: Plotted on the left are the relative frequencies of states sorted per day for each cluster. Plotted on the right are the 10 most typical pathways for each cluster by highest neighborhood density bottom up according to their representative score and a bar width proportional to the number of assigned sequences. The neighborhood radius (i.e., the percentage of the maximum theoretical distance between two sequences) was set to 10%. Coverage describes how many sequences are represented by the 10 most typical pathways

Figure 1 shows the relative frequency of sorted states on each day of the follow-up period as well as the 10 most typical pathways of each cluster. The representativeness of these typical pathways is shown by the height of the bar width, which is proportional to the represented sequences and the coverage.

In Cluster 0, the most typical pathways only show the index episode, consisting of either an inpatient episode with or without QWT. In Cluster 1, postacute treatment was typically not utilized during the follow-up period. The most representative sequence is a short index episode followed by a longer inpatient episode within the first month of the follow-up period. Further representative sequences have no clear pattern of timing of subsequent treatment after the index episode. One representative pathway shows up to three QWTs within the follow-up period. In Cluster 2, rehabilitation treatments are mainly within day 40 and day 163 of the follow-up period, which cannot always be explained by a longer or shorter index episode (see Additional file 1: Fig. S2). In Cluster 2, the few patients additionally using pharmacotherapy for relapse prevention did so some time after finishing their rehabilitation treatment directly following an inpatient episode. Cluster 3 shows several hospital episodes within the follow-up period, but each one is rather short, showing no pattern of timing in the follow-up period. One pathway had already started pharmacotherapy before the index episode.

### Patient characteristics by cluster

Table 2 shows the results of the multinomial logistic regression based on a sample of 514 patients. Four patients from Cluster 1 (*n* = 2) and Cluster 2 (*n* = 2) were excluded from this analysis due to missing data on nationality. The multivariate regression showed no significant differences concerning sex, which were significant in the univariate analysis (*p *= 0.044, see Table 1).

**Table 2:** Multinomial logistic regression for cluster membership

	Cluster 1^**1**^		Cluster 2^**1**^		Cluster 3^**1**^	
	OR	95%-CI	OR	95%-CI	OR	95%-CI
Female (ref.: Male)	0.99	0.64 – 1.54	0.15	0.02 – 1.12	0.61	0.12 – 3.02
Age (centered)	1.00	0.98 – 1.01	0.97	0.94 – 1.01	0.99	0.93 – 1.05
Nationality (ref: Not German)	1.10	0.58 – 2.10	0.83	0.21 – 3.25	0.87	0.10 – 7.42
Comorbidity Score	1.00	0.97 – 1.03	1.03	0.98 – 1.10	1.03	0.94 – 1.14
Inpatient Episode (not F10.2-4) before Index-Episode	1.15	0.67 – 1.98	1.77	0.62 – 5.02	**0.00**	**0.00 – 0.00**
% Hospital days due to Alcohol Dependence (F10.2-4) in follow-up period	1.00	1.00 – 1.01	1.01	0.99 – 1.03	1.04	0.99 – 1.10
Outpatient addiction care 60 days before Index Episode	1.39	0.50 – 3.88	0.42	0.07 – 2.59	**0.00**	**0.00 – 0.00**
Outpatient addiction care within follow-up period	**3.82**	**1.75 – 8.35**	**9.35**	**2.95 – 29.62**	3.15	0.34 – 29.03

Regression parameters in bold signify *p* value < 0.001; *OR*, odds ratio; *CI*, 95% confidence interval; ^1^Cluster 0 served as the reference cluster. Based on a sample of 514 patients. Four patients from Cluster 1 (*n* = 2) and Cluster 2 (*n* = 2) were excluded from this analysis due to missing data on nationality

In total, Cluster 0 had the highest share of females of all clusters (26.4%, see Table 1). The smallest share of females was seen in Cluster 2, with 3.8%. Cluster 2 had the lowest mean age (47.5 years), but the differences between all clusters were neither significant overall (*p* = 0.546, see Table 1) nor in the regression model. The other clusters had a similar mean age of approximately 50 years. No differences concerning nationality could be identified. Differences in the comorbidity score were neither significant in univariate analysis (*p* = 0.593, see Table 1) nor in the regression model. However, Cluster 2 had the highest interquartile range (0.0 to 10.3, see Table 2). There were no statistically significant differences concerning hospital episodes not due to alcohol dependence 60 days before the index event or the share of inpatient episodes due to alcohol dependence in the observation period. Both Cluster 1 and Cluster 2 showed a higher usage of outpatient addiction care services than Cluster 0 (see Table 1). In the regression model, this difference was only statistically significant when looking at the follow-up period (see Table 2).

The sensitivity analysis excluding individuals with missing insurance days in the observation period yielded similar results (see Additional file 1: Table S2 and Fig. S4).

## Discussion

The exploratory state sequence analysis presented here shows that after an inpatient episode due to alcohol dependence or withdrawal symptoms, generally, few patients utilized further postacute treatment. The cluster analysis resulted in four clusters. The two largest clusters are best represented by either no further addiction-specific treatment besides the index event or no (seamless) postacute treatments. The two very small clusters are best represented by using rehabilitation or pharmacotherapy as postacute treatment. These clusters of addiction-specific care pathways showed statistically significant differences in the utilization of outpatient addiction care services. However, there were no statistically significant differences in terms of sociodemographic characteristics or general comorbidities. Furthermore, differences in the utilization of treatment and care services could be visualized and described. The most typical care pathway consisted of not using any of the addiction-specific treatment/care services included in this study. The most used addiction-specific treatment was QWT with no further postacute treatment. To a lesser extent, there seemed to be some patients best characterized by multiple inpatient episodes and QWTs without postacute treatment. There also seemed to be a small subgroup of people showing a revolving door phenomenon of QWTs. Studies among people with psychiatric disorders have most consistently identified previous admissions to be connected to higher readmission rates [22, 23]. A Swiss study could additionally identify symptom load at discharge as an important predictor for higher readmission rates for patients with substance use disorder [24]. Other studies showed a high readmission risk for patients with alcohol use disorder, especially if an alcohol-induced psychiatric disorder was present [25, 26].

Current guidelines recommend withdrawal treatment to be followed seamlessly by postacute treatment, i.e., either rehabilitation treatment, pharmacotherapy for relapse prevention, or other types of postacute treatments [5]. Only two very small clusters of pathways utilize postacute treatments. If a QWT was utilized before a rehabilitation treatment, patients had a waiting period of under 23 days in the median. This indicates quite quick transfers in most cases, nevertheless, leaves room for further reductions of waiting periods. In a nationwide survey of hospital personnel in 81 clinics that provided QWT in 2013, long waiting times for rehabilitation and psychiatric postacute treatments were significant obstacles for seamless referrals. Additionally, clarification of cost coverage and insufficient specific treatment options for patients with severe comorbidities as well as parents of school-age children were reported major challenges [27]. The follow-up period theoretically encompasses the introduction of new recommendations for action by healthcare providers and payers set in effect in August 2017, with the goal of improving seamless access to medical rehabilitation after QWT [28]. They are specifically set up to improve and increase seamless transferals directly from the hospital to rehabilitation treatment. In our data, however, sequences started in most cases well before August 2017, and the effects were likely not yet visible. In the total sample, only 30% of patients utilized QWT, and overall, the usage of postacute treatments was rather low, with only 9.3% using either pharmacotherapy for relapse prevention (3.5%) or rehabilitation (6.4%).

These results cannot be generalized to all of Germany but rather represent the city of Bremen in northern Germany. However, rates of addiction-specific treatment utilization in the general population in Bremen based on the total sample of the used data set were found to be comparable to German-wide estimates [13]. In the present study, the proportion of patients utilizing pharmacotherapy, i.e., acamprosate, disulfiram, or naltrexone, was 3.5%. These results are in line with another analysis of routine data in northern Germany concluding an underutilization of pharmacotherapy as postacute treatment, i.e., acamprosate and naltrexone [29]. Only 2.2% of patients in the six months following an inpatient treatment due to alcohol dependence (F10.2) or withdrawal state (F10.3–4) received this kind of anti-craving medication [29]. An increase in pharmacotherapy for relapse prevention may have desirable effects on hospitalization rates among people with alcohol use disorders [30]. A study from the UK evaluated nalmefene, in combination with psychosocial support, as a cost-efficient treatment option for a population with high drinking risk levels but without the need for immediate detoxification [31]. This shows that pharmacotherapy for relapse prevention may not be suitable for every individual with alcohol dependence but poses an option in specific cases. Therefore, increasing the utilization may be beneficial. However, more comparable studies are needed to formulate generally effective strategies to achieve this, as a review concluded [32].

The utilization of other types of postacute treatments was not documented in the analyzed data sets. It can be assumed that not everyone with a diagnosis of alcohol dependence needs the kind of postacute treatments analyzed here, i.e., pharmacotherapy for relapse prevention or a rehabilitation treatment. However, there is robust evidence that postacute treatments are beneficial for those who need them. Timely access of patients to addiction-specific care/treatment after withdrawal reduces readmissions and can be seen as a “teachable moment” for patients to increase their motivation to engage in further treatment [5]. Studies from the USA and Canada indicate higher initial engagement and reduced readmissions when withdrawal treatment is followed by seamless postacute care [33, 34]. In particular, early rehabilitation (within three months of detoxification) appears to be beneficial in reducing the likelihood of relapse [35]. One possible way to improve referrals to postacute treatment may be patient-centered placement matching approaches using a standardized assessment of the level of care needed for patients after a QWT [36]. In a randomized control trial in four German psychiatric clinics analyzing patients following an inpatient QWT due to alcohol dependence without organized aftercare, reductions in days of heavy drinking and lowering of costs were identified as benefits, whereas the actual referral to aftercare remained unchanged [37]. This indicates the need for more research on how to increase the number of people who utilize the recommended postacute treatment.

Since 2010, there has been a negative trend in the utilization of rehabilitation treatments overall, particularly for addiction treatment [38]. Reasons were structural barriers like the high bureaucratic effort required for applications, low expectations of successful outcomes, and insufficient information about available rehabilitations [38]. Beside these structural barriers a general improvement in outpatient offers of addiction-specific postacute care and outpatient psychotherapeutic measures [38], as well as a decline in alcohol use, alcohol dependence, and alcohol-related morbidity and mortality indicate an overall lower demand for rehabilitation treatments [39, 40]. Future analyses should therefore include more outpatient postacute treatments. However, alcohol dependence is highly stigmatized, and research has shown that higher levels of stigma are associated with reduced help-seeking behavior in general [41]. Also, patient-related barriers are relevant, such as a lack of problem awareness, a desire to continue drinking, and a preference to handle the issue independently. Interestingly, problem awareness appears to be negatively correlated with the severity of dependence [42].

This study focused on people in a more severe stage of their dependence as only patients with at least one inpatient episode were analyzed. Hospital episodes appear to help facilitate motivation for further addiction-specific treatment. Patients with alcohol dependence who were admitted to a hospital show a higher motivation to change than people with alcohol dependence in the general population [43]. In our study, people with a higher number of hospital days utilized more addiction-specific care services. However, pharmacotherapy for relapse prevention was the only addiction-specific treatment that could be identified in the outpatient setting, in which more than half of the individuals with alcohol dependence are estimated to already be recognized [13]. Additionally, only inpatient QWTs could be identified, which under specific circumstances can also take place in outpatient settings. Hence, the number of total QWTs is underestimated.

The data set also only contains rehabilitation data from one of three German Pension Insurances (DRV), namely the regional DRV Bremen/Oldenburg, and is missing rehabilitation treatments financed by health insurances (Germany-wide around 15% of all inpatient rehabilitation treatments in specialized clinics for alcohol and drug dependence [44]) and the other German Pension Insurances, i.e., federal DRV Bund and DRV Knappschaft-Bahn-See. Of all approved rehabilitation services financed by the German Pension Insurance in Bremen in 2016/2017 66% were financed by the regional DRV Bremen/Oldenburg, 29% by the federal DRV Bund, and 5% by the DRV Knappschaft-Bahn-See (Deutsche Rentenversicherung Bund, unpublished data, 2024). Hence, the total number of utilized postacute treatments is underestimated. In a randomized control trial study of patients with alcohol-use disorder in eight clinics in southern Germany, postacute care was used by almost half of the sample following inpatient withdrawal management (with and without QWT) without organized after care [35]. Outpatient addiction care (22–37%) and self-help groups (7–15%) were the predominant postacute care services utilized, alongside rehabilitation, while psychiatric treatment was not reported [35].

### Strengths and limitations

The primary strength of this paper is the data linkage of routine data of several addiction-specific care providers for inpatient and outpatient services as well as rehabilitation treatment services. This approach mitigates potential biases typically associated with surveys, such as response or memory bias. Additionally, the insurance data covers all services utilized by people living in Bremen and is therefore except for the outpatient addiction care data of the communal hospital group not reduced on services taking place in Bremen. Additionally, since data from the pension funds was used, rehabilitation treatments were also independent of location and setting of the treatment. Furthermore, the present data set represents a large part of the highly fragmented care system for people with alcohol dependence in Germany.

Nevertheless, there are some drawbacks. Specific areas of addiction-specific help are not part of the data set, for example, self-help therapy groups. Furthermore, the analysis is missing additional addiction-specific care services and treatments in the outpatient setting, e.g., brief interventions and psychotherapy. These services could not be identified since necessary codes, like uniform assessment standard (Einheitlicher Bewertungsmaßstab (EBM)), were not part of the data set. Also, outpatient addiction care outside of the communal hospital group is not part of the data. Utilization of addiction-specific treatments and care services, in general, relies on different variables, such as drinking patterns or severity of dependence, which could not be measured in the present data.

The analyzed population of the statutory heath insurances show similar age and gender distribution compared to the total population [13]. Still, systematic differences regarding addiction-specific care pathways cannot be ruled out, since important variables associated with health care utilization can differ between health insurance funds. Earlier studies using AOK data for Lower Saxony, which is the state surrounding Bremen, showed an overrepresentation of people with a lower socio-economic status and migration background compared to the total population [45]. At the same time, there is a higher share of people with a high socio-economic status in private health insurance funds (requirements: earning more than the compulsory insurance threshold, being a public servant or self-employed), and other funds than AOK [46]. To mitigate biases in this study also data from the hkk was integrated. If possible, data from all insurance funds should ideally be used in future studies. A higher utilization of addiction-specific treatments seems unlikely, as e.g., private health insurance companies, in which only approx. 10% are insured in Germany [47], do not always cover addiction specific treatments like QWT.

The cluster sizes in the present analysis are very small; however, the cluster solution presented shows an average silhouette width above 0.5, which can be described as a reasonable structure [48]. When analyzing the clusters graphically, several differences in the usage of care services and patterns of utilization were visible. The small cluster sizes are mostly due to the low number of sequences that used addiction-specific treatment and the rather heterogeneous sample. Additionally, the cluster solution was not robust. When setting the effect window of the pharmacotherapy to 60 days, all cluster solutions had an average silhouette width under 0.5 and therefore could be described as weak and potentially artificial (data not shown).

The index event and the pre-index period were selected to obtain a homogenous sample with a comparable severity of addiction and a comparable stage of treatment. Nevertheless, some pathways already showed postacute treatment in the earliest quarters following an inpatient episode or pharmacotherapy after rehabilitation treatment. This suggests that patients could have been in different stages of their addiction. The data are both right- and left-censored, which makes it difficult to define a follow-up time that is long enough and still having a comparable sample. In particular, alcohol dependence is characterized by a long time gap between the onset of symptoms and treatment seeking [44] and a high readmission risk. Future studies should consider a longer observation period to be able to analyze long-term care pathways and have a large enough sample to be able to define more homogenous groups.

## Conclusions

The state sequence analysis showed that even when addiction-specific care services are utilized, only a minority use postacute treatments after QWT, i.e., rehabilitation treatment or pharmacotherapy for relapse prevention. Even though the cluster solution was not very robust and cluster sizes were small, different patterns of utilizing addiction-specific treatment and care services, specifically concerning postacute treatments, could be presented. The different patterns of utilization could not be explained by differences in sociodemographic characteristics or general comorbidity. Although using QWT and therefore being in contact with addiction-specific care networks, many people either seek treatment after withdrawal outside the healthcare system or do not utilize further addiction-specific care services at all. Therefore, not only is the generally low utilization of addiction-specific care services of concern but also the low utilization of postacute treatments in the care pathway of individuals after withdrawal treatment despite being recommended by the current treatment guidelines.

### Supplementary Information

Supplementary Material 1.

Supplementary Material 2.

## Data Availability

The IMPELA dataset used and/or analyzed during the current study is available on reasonable request. Requests to access these datasets should be directed to https://www.impela.de/kontakt/.

## Abbreviations

ATC: Anatomical-therapeutical-chemical classification
ICD: International Classification of Diseases
OPS: Surgery- and Procedure-Code (Operationen- und Prozeduren-Schlüssel)
QWT: Qualified withdrawal treatment
