# Supplementary material for: Clustering care pathways of people with alcohol dependence using a data linkage of routine data in Bremen, Germany
Source: BMC Med. 2024 May 30;22:219. doi: 10.1186/s12916-024-03438-4 (PMC11140874; doi:10.1186/s12916-024-03438-4)
Supplement: Supplementary file 1 — Supplementary Material 1. [file 12916_2024_3438_MOESM1_ESM.docx]

**Additional File 1:**

**Table S1: Overview of alcohol-related ICD-10 diagnoses**

| ICD -10 Codes | Meaning according to ICD-10 |
| --- | --- |
| F10 | Mental and behavioral disorders due to use of alcohol (includes F10.1 - F10.9) |
| F10.0 | Acute intoxication |
| F10.1 | Harmful use |
| F10.2 | Dependence syndrome |
| F10.3 | Withdrawal state |
| F10.4 | Withdrawal state with delirium |
| F10.5 | Psychotic disorder |
| F10.6 | Amnesic syndrome |
| F10.7 | Residual and late-onset psychotic disorder |
| F10.8 | Other mental and behavioral disorders |
| F10.9 | Unspecified mental and behavioral disorder |
| E24.4 | Alcohol-induced pseudo-Cushing syndrome |
| E52 | Niacin deficiency [pellagra] |
| G31.2 | Degeneration of nervous system due to alcohol |
| G62.1 | Alcoholic polyneuropathy |
| G72.1 | Alcoholic myopathy |
| I42.6 | Alcoholic cardiomyopathy |
| K29.2 | Alcoholic gastritis |
| K70.- | Alcoholic liver disease |
| K85.2 | Alcohol-induced acute pancreatitis |
| K86.0 | Alcohol-induced chronic pancreatitis |
| O35.4 | Maternal care for (suspected) damage to fetus from alcohol |
| P04.3 | Fetus and newborn affected by maternal use of alcohol |
| Q86.0 | Fetal alcohol syndrome (dysmorphic) |
| R78.0 | Finding of alcohol in blood |
| T51.- | Toxic effect of alcohol |
| T51.0 | Ethanol |
| T51.9 | Alcohol, unspecified |

Table S2: Sensitivity Analysis of multinomial logistic regression for cluster membership using only individuals without missing insurance days

|  | **Cluster 1^1^** | | **Cluster 2^1^** | | | | **Cluster 3^1^** | |
| --- | --- | --- | --- | --- | --- | --- | --- | --- |
|  | **OR** | **95%-CI** | | **OR** | **95%-CI** | **OR** | | **95%-CI** |
| Female (ref.: Male) | 0.96 | 0.62 – 1.51 | | 0.16 | 0.02 – 1.22 | 0.62 | | 0.13 – 3.04 |
| Age (centered) | 1.00 | 0.98 – 1.01 | | 0.97 | 0.93 – 1.01 | 0.99 | | 0.93 – 1.05 |
| Nationality (ref: Not German) | 0.99 | 0.51 – 1.92 | | 0.52 | 0.10 – 2.60 | 0.81 | | 0.09 – 6.93 |
| Comorbidity Score | 1.00 | 0.97 – 1.03 | | 1.02 | 0.96 – 1.08 | 1.03 | | 0.93 – 1.13 |
| Inpatient Episode (not F10.2-4) before Index-Episode | 1.10 | 0.63 – 1.91 | | 2.33 | 0.80 – 6.81 | **0.00** | | **0.00 – 0.00** |
| % Hospital days due to Alcohol Dependence (F10.2-4) in follow-up period | 1.00 | 1.00 – 1.01 | | 1.02 | 1.00 – 1.05 | 1.04 | | 0.99 – 1.10 |
| Outpatient addiction care 60 days before Index Episode | 1.22 | 0.43 – 3.44 | | 0.41 | 0.06 – 2.63 | **0.00** | | **0.00 – 0.00** |
| Outpatient addiction care within follow-up period | **4.62** | **2.06 – 10.35** | | **11.25** | **3.23 – 39.21** | 3.69 | | 0.39 – 34.58 |
| Notes: Regression parameters in bold signify p value<0.001; OR: Odds-Ratio; CI: 95%-Confidence Interval; ^1^ Cluster 0 served as the reference cluster. Based on a sample of 498 patients. Four patients from Cluster 1 (n=2) and Cluster 2 (n=2) were excluded from this analysis due to missing data on nationality and 16 patients due to missing insurance days within the observation period. | | | | | | | | |

Figure S1: Month of first inpatient episode due to alcohol dependence (F10.2) or withdrawal (F10.3-4) in the total sample


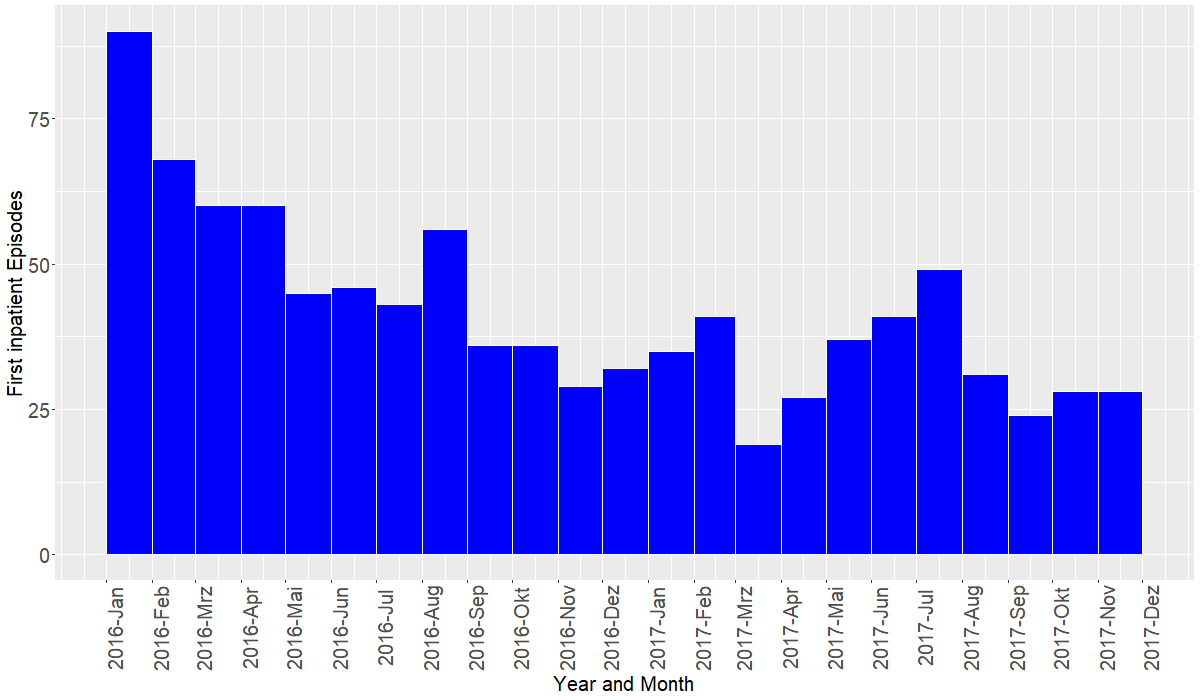


Figure S2: Sequences of addiction-specific care services after an inpatient episode with main diagnosis alcohol dependence (F10.2-4) total and by clusters
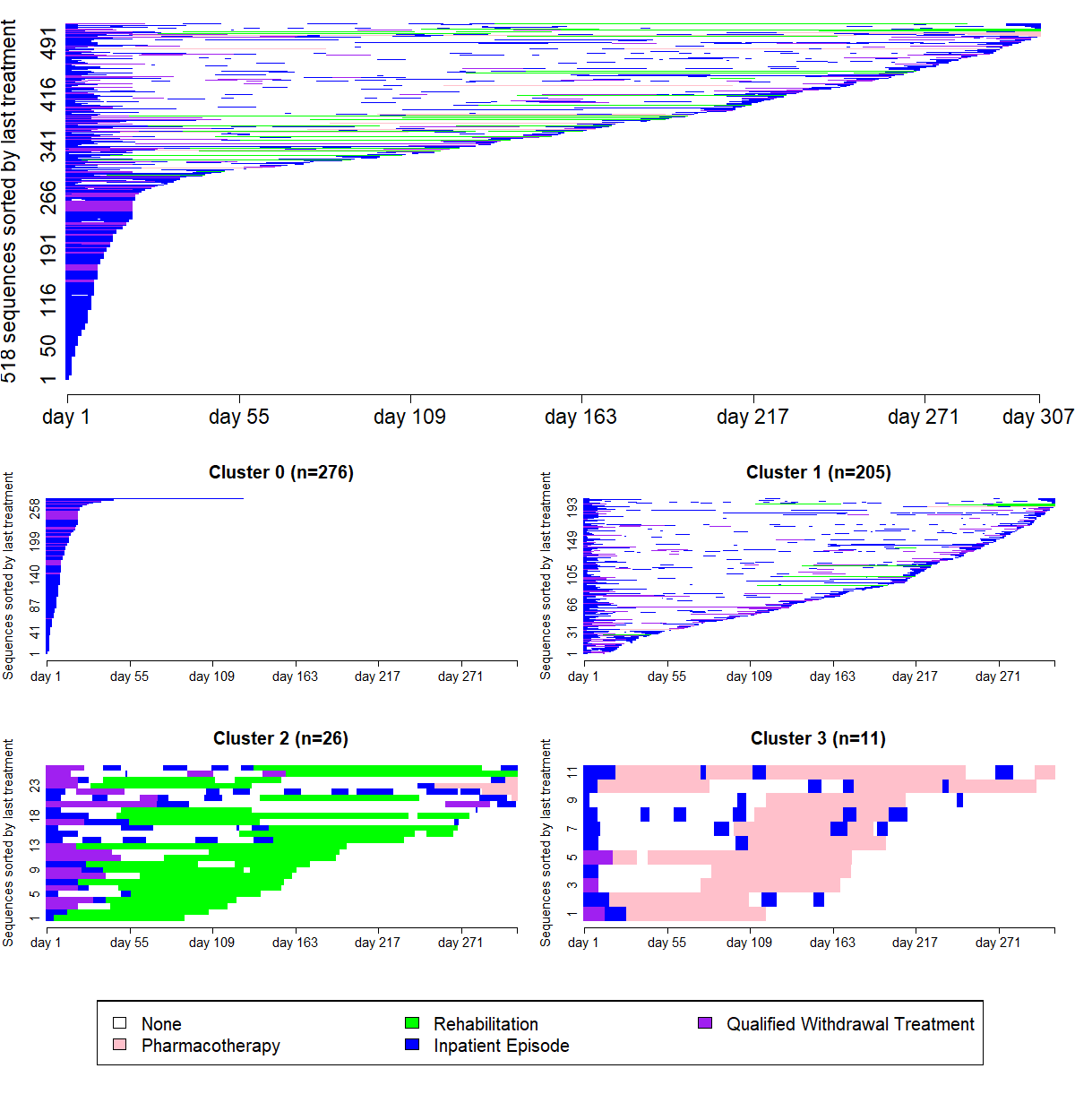


Figure S3: Days between the end of qualified withdrawal treatment and the onset of rehabilitation by cluster


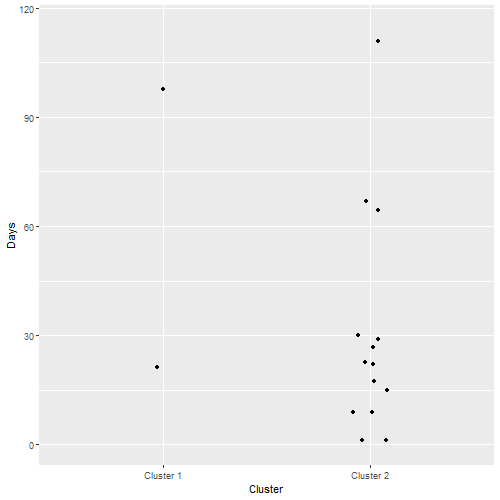


Figure S4: Sensitivity Analysis for sequences of addiction-specific care services after an inpatient episode with main diagnosis alcohol dependence (F10.2-4) total and by clusters without missing insurance days


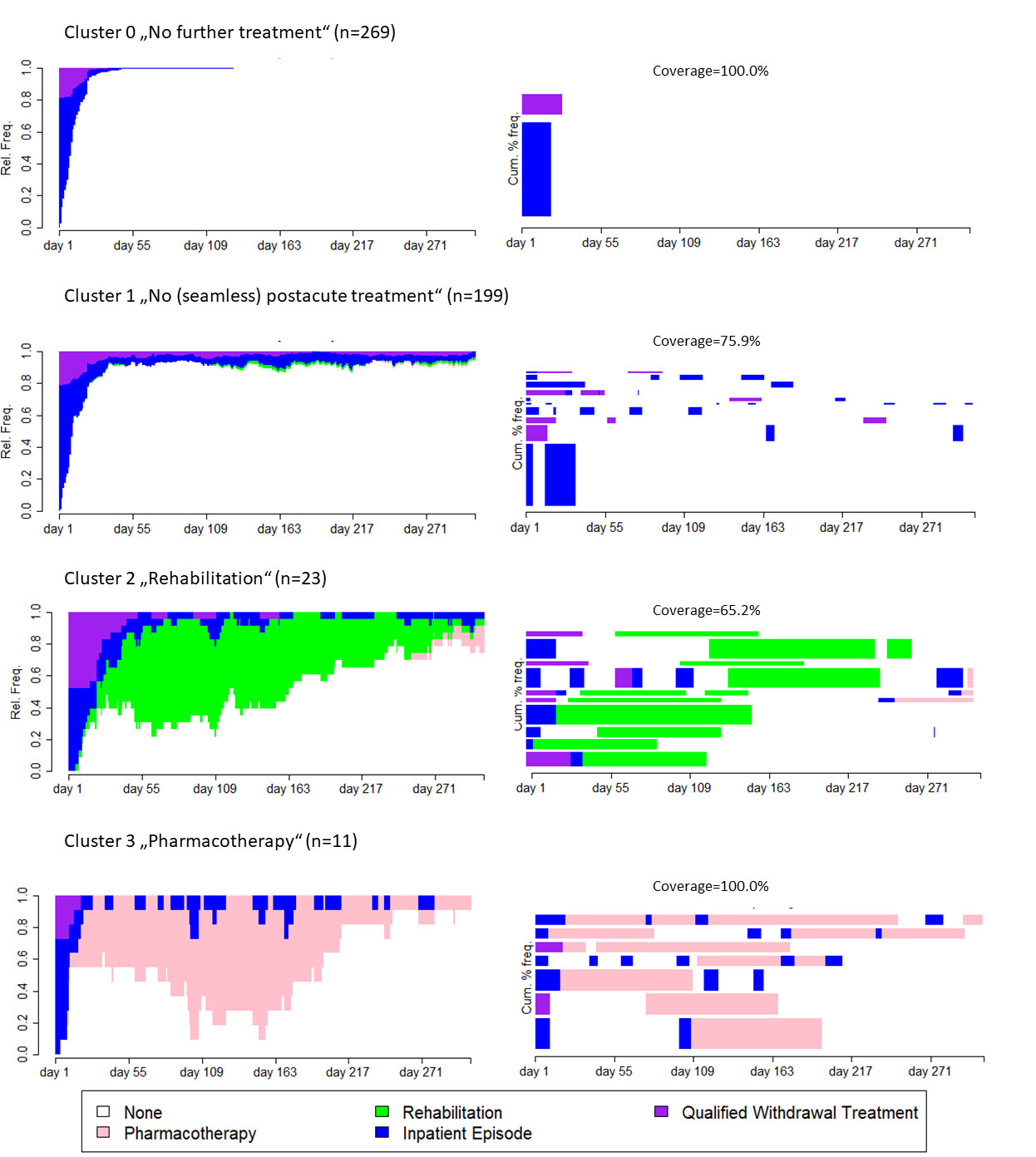


Notes: Plotted on the left are the relative frequencies of states sorted per day for each cluster. Plotted on the right are the 10 most typical pathways for each cluster by highest neighborhood density bottom up according to their representative score and a bar width proportional to the number of assigned sequences. The neighborhood radius (i.e., the percentage of the maximum theoretical distance between two sequences) was set to 10%. Coverage describes how many sequences are represented by the 10 most typical pathways.
